# Locally advanced port-site metastasis following laparoscopic surgery for early-stage endometrial carcinoma: a case report

**DOI:** 10.1093/jscr/rjaf509

**Published:** 2025-07-14

**Authors:** Sabrillah Echiguer, Soumaya Elgraini, Yassine El Bouazizi, Fatima Zahra Belkouchi, Fouad Tijami, Youssef Omor, Rachida Latib, Hafid Hachi, Nezha El Bahaoui

**Affiliations:** Department of Surgical Oncology, National Institute of Oncology, Faculty of Medicine and Pharmacy, Mohammed V University in Rabat, Morocco; Department of Radiology, National Institute of Oncology, Faculty of Medicine and Pharmacy, Mohammed V University in Rabat, Morocco; Department of Surgical Oncology, National Institute of Oncology, Faculty of Medicine and Pharmacy, Mohammed V University in Rabat, Morocco; Department of Surgical Oncology, National Institute of Oncology, Faculty of Medicine and Pharmacy, Mohammed V University in Rabat, Morocco; Department of Surgical Oncology, National Institute of Oncology, Faculty of Medicine and Pharmacy, Mohammed V University in Rabat, Morocco; Department of Radiology, National Institute of Oncology, Faculty of Medicine and Pharmacy, Mohammed V University in Rabat, Morocco; Department of Radiology, National Institute of Oncology, Faculty of Medicine and Pharmacy, Mohammed V University in Rabat, Morocco; Department of Surgical Oncology, National Institute of Oncology, Faculty of Medicine and Pharmacy, Mohammed V University in Rabat, Morocco; Department of Surgical Oncology, National Institute of Oncology, Faculty of Medicine and Pharmacy, Mohammed V University in Rabat, Morocco

**Keywords:** endometrial cancer, laparoscopic surgery, port-site metastasis, case report

## Abstract

Port-site metastasis (PSM) following minimally invasive surgery for early-stage endometrial cancer is rare, and its underlying pathophysiology remains poorly understood. Currently, no standardized treatment protocol exists. We report the case of a 66-year-old woman who developed a locally advanced PSM 18 months after undergoing laparoscopic surgery for early-stage endometrioid adenocarcinoma. She was treated with neoadjuvant chemotherapy to reduce the mass and enable complete surgical resection. Although the incidence of PSM is low, its management poses significant clinical challenges and can be associated with a poor prognosis. Early recognition, an adapted multidisciplinary approach, and implementation of preventive strategies during initial surgery are critical to optimizing outcomes.

## Introduction

Endometrial cancer (EC) is the sixth most common cancer in the world [1]. The standard surgical treatment for early EC is total hysterectomy, bilateral salpingo-oophorectomy, and surgical staging [2]. Traditionally, laparotomy was the conventional surgical approach for EC. Nowadays, minimally invasive surgery is preferred for early stages [2]; it reduces operative morbidity and hospital stay, with comparable survival rates to laparotomy [3]. Port-site metastasis (PSM) is defined by cancer growth at the site of a port incision after laparoscopic resection of a malignant tumor, and by definition, it’s not associated with diffuse peritoneal carcinomatosis [4]. PSM has also been reported in patients with early EC [5]. Even though the risk remains low, the treatment can be challenging, and the prognosis can be severe. We report a case of locally advanced isolated PSM following laparoscopic surgery for early EC, and the difficulty of its management.

## Case report

A 66-year-old nulliparous woman presented with postmenopausal vaginal bleeding to her gynecologist. The endometrial biopsy revealed complex endometrial hyperplasia with atypia. She subsequently underwent total laparoscopic hysterectomy and bilateral salpingo-oophorectomy. The final hysterectomy specimen revealed a grade 2 endometrioid endometrial adenocarcinoma, measuring 3.5 cm in maximum diameter, infiltrating more than half of the myometrium, and invading the cervix (pT2). The patient was referred to our center for further management. The multidisciplinary team meeting (MDT) decided to proceed with external radiotherapy.

**Figure 1:**
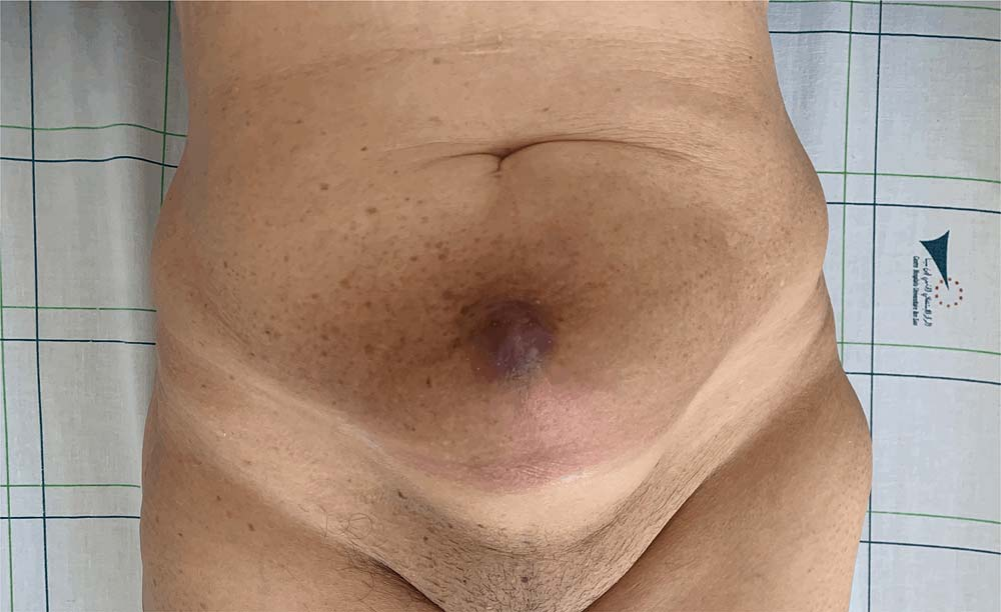
Mass at the previous trocar site with inflammatory signs.

**Figure 2:**
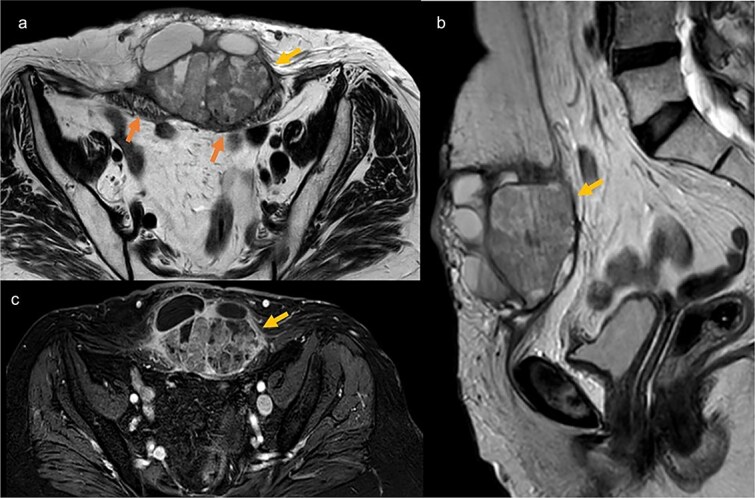
Pelvic MRI in axial T2 (a), sagittal T2 (b), and axial T1 post-contrast (c), showing a well-defined mass at the trocar site, infiltrating the rectus abdominis muscles. The mass has regular contours, heterogeneous content with both fluid and tissue components, and exhibits enhancement after contrast injection.

Eighteen months later, surveillance revealed a large mass of 12 cm at hypogastric trocar orifice, invading the skin with inflammatory signs (Fig. 1). The pelvic MRI revealed a heterogeneous lesion in the anterior abdominal wall, centered on the left rectus abdominis muscle, measuring 86 × 59 × 75 mm, suggestive of tumor recurrence (Fig. 2). PET scan showed a hypermetabolic pathological pelvic wall mass extending to the left side with a suspicious appearance. No other suspicious metabolic anomalies were detected in the visceral organs or bones. Ultrasound-guided biopsy revealed a moderately differentiated adenocarcinoma with an immunohistochemical profile compatible with endometrial origin.

The decision at the MDT was to initiate first-line chemotherapy with Paclitaxel and Carboplatin, due to the size of the mass, which could complicate abdominal wall closure after tumor resection, and also considering the presence of inflammatory signs.

**Figure 3:**
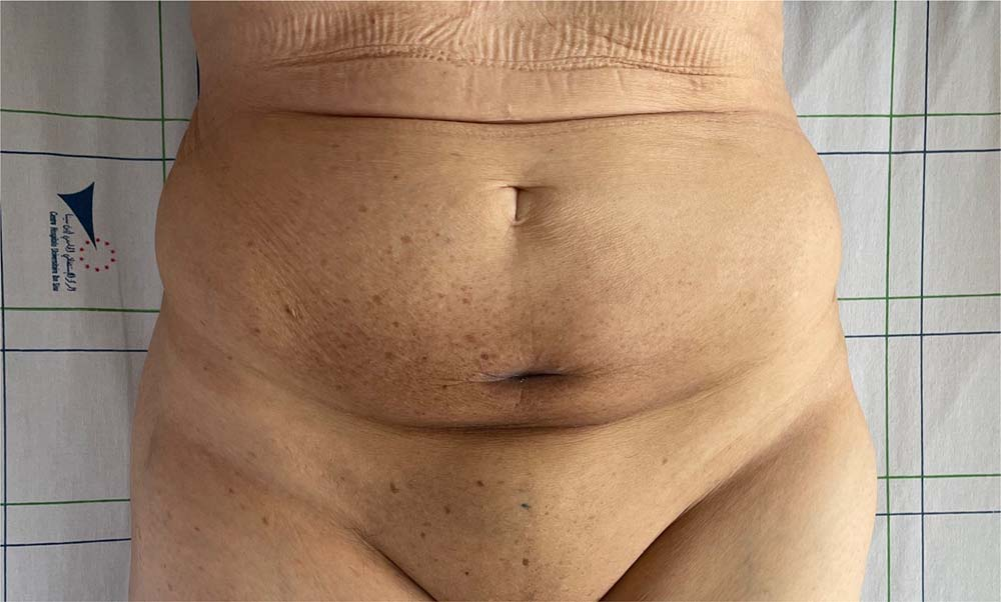
Clinical regression of the mass and disappearance of inflammatory signs after the third cycle of chemotherapy.

**Figure 4:**
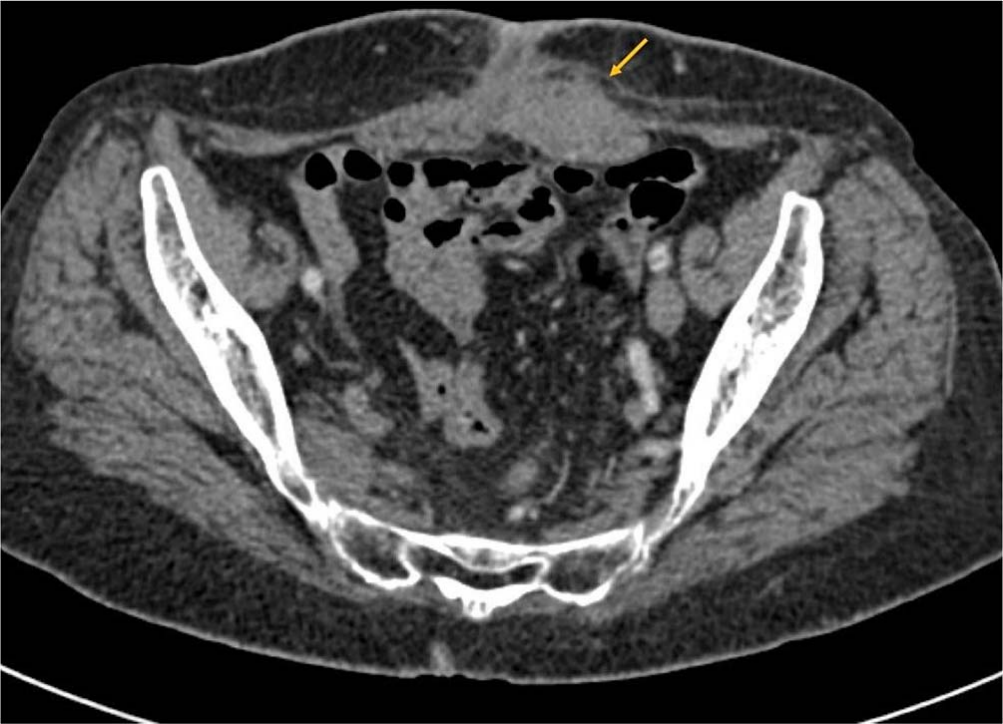
Axial CT scan showing a significant reduction in the size of the mass post-chemotherapy, now confined to the left rectus abdominis muscle with fat infiltration.

The patient received six cycles of Paclitaxel and Carboplatin, with a good clinical and radiological response (Figs 3 and 4). A follow-up computed tomography (CT) scan after the 3rd cycle of chemotherapy showed a significant reduction in tumor size, measuring 66 × 50 × 30 mm compared to 86 × 59 × 75 mm (Fig. 4).

**Figure 5:**
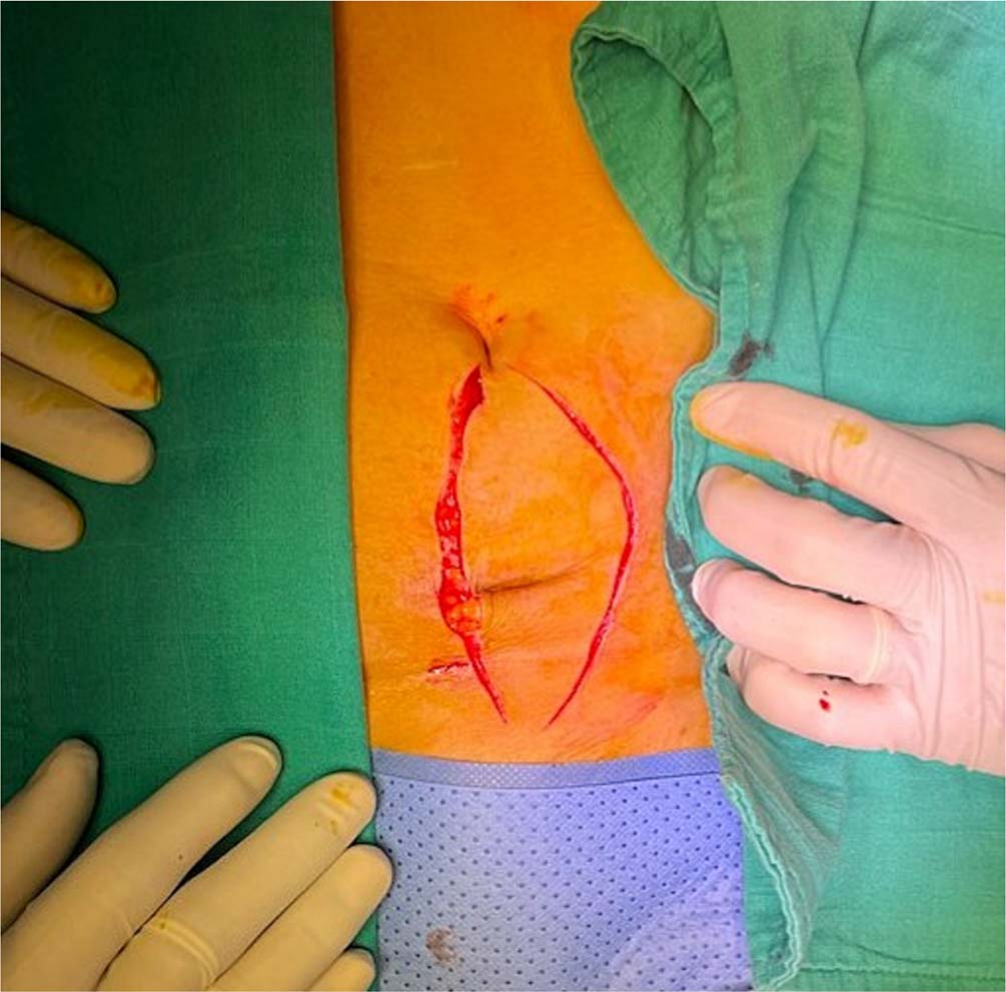
Initial skin incision. The incision was later extended on the left to ensure adequate safety margins.

**Figure 6:**
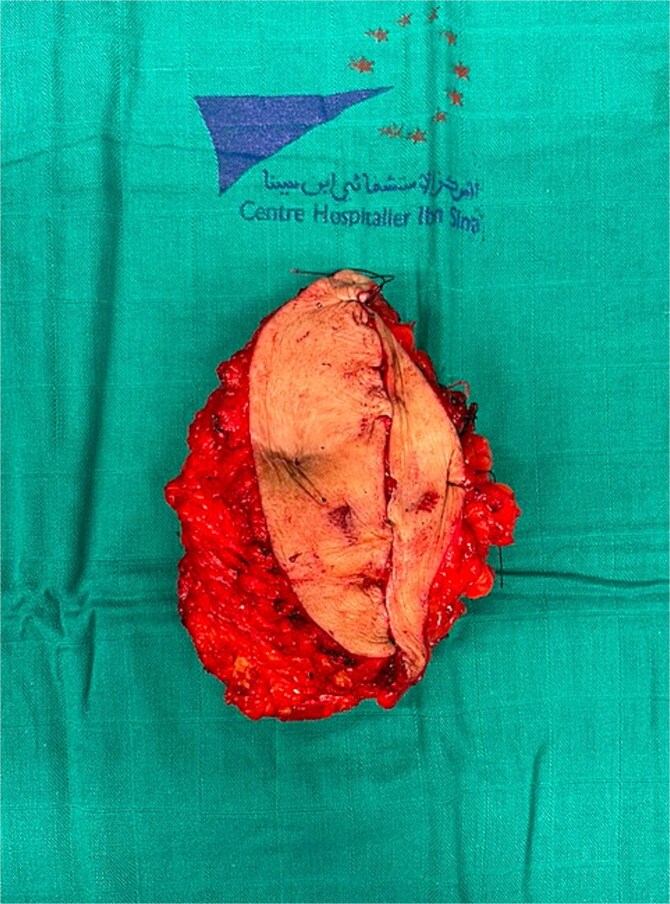
Surgical specimen.

**Figure 7:**
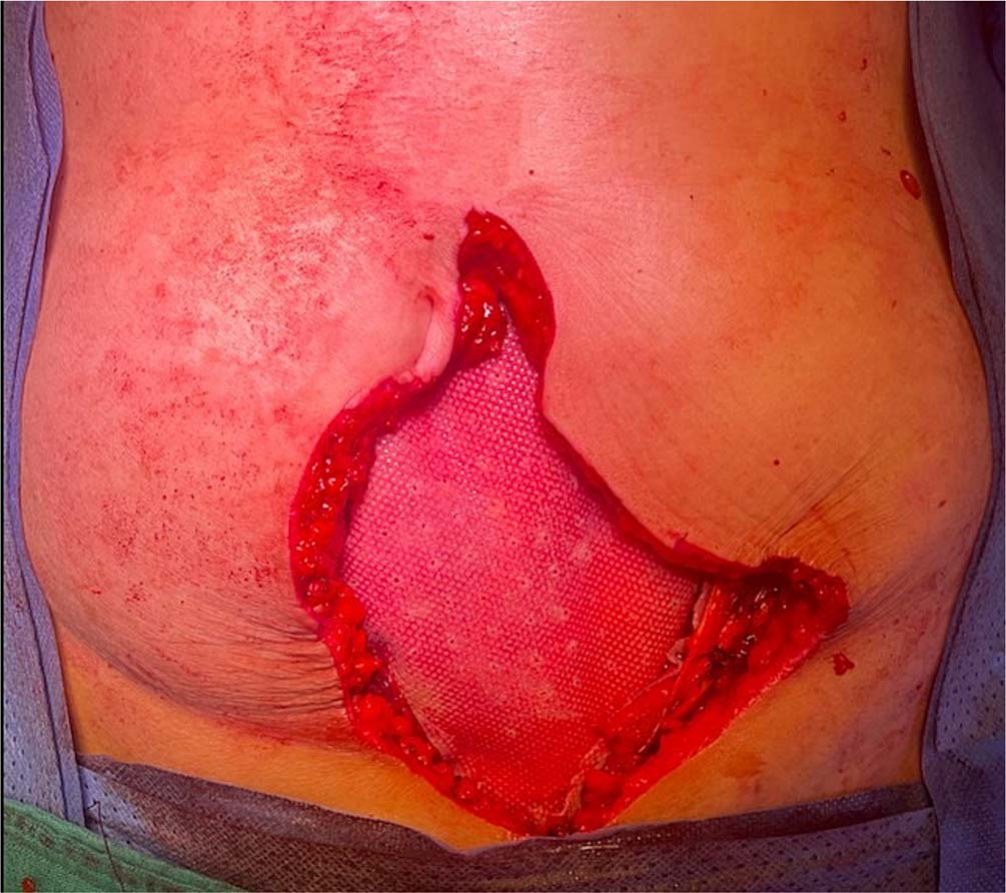
Abdominal wall reconstruction with a dual mesh.

**Figure 8:**
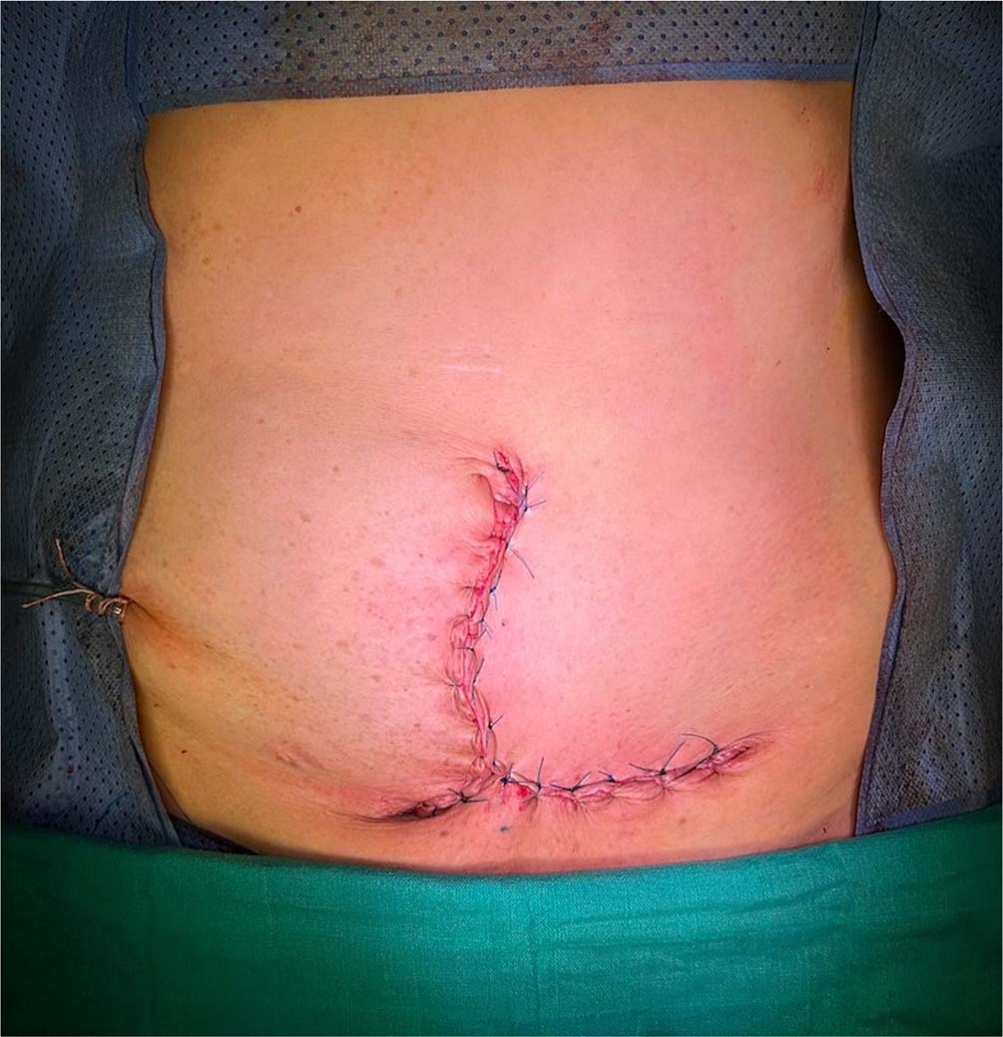
Appearance of the abdominal scar following the procedure.

The patient underwent surgery 3 weeks after the 6th chemotherapy cycle. An elliptical skin incision was made on both sides of the mass (Fig. 5). The mass was dissected and resected with clear macroscopic margins (Fig. 6). Surgical exploration revealed no intraperitoneal tumor extension. No local recurrence, peritoneal carcinomatosis, or hepatic metastasis was found. Due to the large defect, parietal closure was achieved using a Biface plate (Fig. 7). A Redon drain was positioned adjacent to the plate. The skin was closed without tension, fully covering the plate, resulting in an inverted T-shaped appearance. (Fig. 8).

The postoperative course was uncomplicated, and the patient was discharged on postoperative day 5. The histopathological examination identifies a post-therapeutic residual carcinomatous lesion, largely necrotic, consistent with the endometrioid carcinoma subtype previously diagnosed in the patient. The surgical margins are free of malignancy. The decision of the MDT is to proceed with surveillance. Follow-up was scheduled every three months during the first year. Currently, at the six-month follow-up visit, clinical examination and thoraco-abdomino-pelvic CT imaging showed no evidence of local or distant recurrence.

## Discussion

Historically, the first case of PSM after laparoscopic surgery for EC was reported in 1997 by Kadar [6]. Subsequently, several cases were reported in the literature, including some after laparoscopic surgery for early EC [5], and also following robotic surgery [7].

Despite multiple cases reported in the literature, the risk of developing PSM remains low. According to the prospective findings from the Gynecologic Oncology Group trial 2222, the incidence of PSM after laparoscopic surgery for EC was 0.24%, with four cases identified among 1696 patients [8].

The etiology of PSM remains unknown in general. Ramirez *et al.* reported several factors identified as contributors to PSM, including wound implantation caused by the surgical technique and instrumentation, the leakage of insufflation gas through the ports, known as the “chimney effect,” and the impact of pneumoperitoneum on local immune reactions [9].

Regarding treatment, there is no consensus in the literature. Palomba *et al.* suggested that excision followed by radiotherapy could be an adequate treatment for isolated PSM. However, the role of chemotherapy remains unclear in such cases [10]. Grant *et al.*, in a retrospective observational cohort study, also reported favorable outcomes for isolated PSM treated with surgical resection and radiotherapy, with or without the addition of chemotherapy [11]. Raffone *et al.,* in a systematic literature review, concluded that resection and radiotherapy with or without chemotherapy seems to be the most appropriate treatment for EC women with PSM [12]. For our patient, the Multidisciplinary Consultation Meeting decided to start first-line chemotherapy with Paclitaxel and Carboplatin, followed by surgical resection, considering the presence of inflammatory signs, and especially the size of the mass that can make the abdominal wall closure more difficult after tumor resection. The response to chemotherapy was remarkable, and the surgery was successfully performed. Concerning the surgical treatment, we used a dual mesh for abdominal wall closure due to the size of the parietal defect following the mass resection. However, skin closure did not require the use of a coverage flap.

Some preventive measures have been described to reduce the risk of PSM, including rinsing trocars with 5% povidone-iodine before insertion, using protective bags to remove the tumor, deflating the abdomen with trocars in place, and irrigating trocar sites with 5% povidone-iodine [9].

Finally, PSM are rare following laparoscopic surgery for early EC and their etiology remains unclear and is likely multifactorial. This case demonstrates that managing locally advanced cases can be challenging, and it is important to focus on preventing PSMs and closely monitoring patients.
